# 1,3-Dicyclo­hexyl­imidazolidine-2,4,5-trione

**DOI:** 10.1107/S1600536811046253

**Published:** 2011-11-09

**Authors:** Oualid Talhi, José A. Fernandes, Diana C. G. A. Pinto, Artur M. S. Silva, Filipe A. Almeida Paz

**Affiliations:** a Department of Chemistry, University of Aveiro, QOPNA, 3810-193 Aveiro, Portugal; b Department of Chemistry, University of Aveiro, CICECO, 3810-193 Aveiro, Portugal

## Abstract

The title compound, C_15_H_22_N_2_O_3_, has been isolated as a by-product of an oxidative cleavage of the C—C bond linking two five-membered rings of 1,3-dicyclo­hexyl-5-(3-oxo-2,3-dihydro­benzofuran-2-yl)imidazolidine-2,4-dione. Individual mol­ecular units are engaged in weak C=O⋯C=O inter­actions [O⋯C = 2.814 (10) and 2.871 (11) Å], leading to the formation of supra­molecular chains which close pack, mediated by van der Waals contacts, in the *bc* plane.

## Related literature

For the synthesis of parabanic acid and its derivatives, see: Murray (1957 ▶, 1963 ▶); Ulrichan & Sayigh (1965 ▶); Richter *et al.* (1984 ▶); Orazi *et al.* (1977 ▶); Zarzyka-Niemiec & Lubczak (2004 ▶). For biological applications of parabanic acid and its derivatives, see: Ishii *et al.* (1991 ▶); Kotani *et al.* (1997 ▶); Sato *et al.* (2011 ▶). For the synthesis, characterization and biological studies of the title compound, see: Xia *et al.* (2011 ▶). For general background to crystallographic studies of compounds having biological activity from our research group, see: Fernandes *et al.* (2010 ▶, 2011 ▶); Loughzail *et al.* (2011 ▶). For the synthesis of a precursor mol­ecule, see: Talhi *et al.* (2011 ▶).
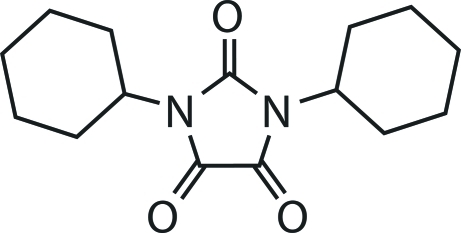


## Experimental

### 

#### Crystal data

C_15_H_22_N_2_O_3_
*M* *_r_* = 278.35
Orthorhombic, 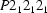

*a* = 6.5539 (8) Å
*b* = 11.5029 (15) Å
*c* = 19.524 (3) Å
*V* = 1471.9 (3) Å^3^
*Z* = 4
Mo *K*α radiation
μ = 0.09 mm^−1^
*T* = 150 K
0.05 × 0.03 × 0.02 mm

#### Data collection

Bruker X8 KappaCCD APEXII diffractometer
Absorption correction: multi-scan (*SADABS*; Sheldrick, 1997 ▶) *T*_min_ = 0.996, *T*_max_ = 0.998
8292 measured reflections
1558 independent reflections
1028 reflections with *I* > 2σ(*I*)
*R*_int_ = 0.047

#### Refinement

*R*[*F* ^2^ > 2σ(*F* ^2^)] = 0.106
*wR*(*F* ^2^) = 0.320
*S* = 1.06
1558 reflections
181 parameters
72 restraints
H-atom parameters constrained
Δρ_max_ = 0.74 e Å^−3^
Δρ_min_ = −0.42 e Å^−3^

### 

Data collection: *APEX2* (Bruker, 2006 ▶); cell refinement: *SAINT-Plus* (Bruker, 2005 ▶); data reduction: *SAINT-Plus*; program(s) used to solve structure: *SHELXTL* (Sheldrick, 2008 ▶); program(s) used to refine structure: *SHELXTL*; molecular graphics: *DIAMOND* (Brandenburg, 2009 ▶); software used to prepare material for publication: *SHELXTL*.

## Supplementary Material

Crystal structure: contains datablock(s) global, I. DOI: 10.1107/S1600536811046253/tk5010sup1.cif

Structure factors: contains datablock(s) I. DOI: 10.1107/S1600536811046253/tk5010Isup2.hkl

Supplementary material file. DOI: 10.1107/S1600536811046253/tk5010Isup3.cml

Additional supplementary materials:  crystallographic information; 3D view; checkCIF report

## Figures and Tables

**Fig. 1.**
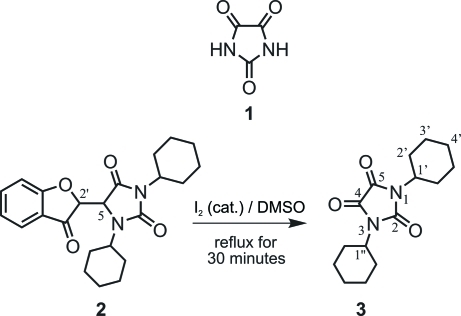
(Top). Molecular representation of imidazolidine-2,4,5-trione (1). (Bottom). Reaction scheme to isolate the title compound (3) from 1,3-dicyclohexyl-(3-oxo-2,3-dihydrobenzofuran-2-yl)imidazolidine-2,4-dione (2).

**Fig. 2.**
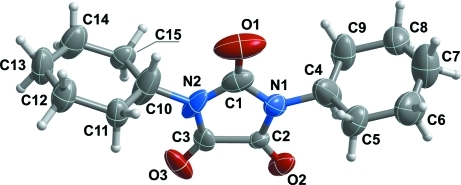
Asymmetric unit of the title compound. Displacement ellipsoids are drawn at the 50% probability level and the atomic labeling is provided for all non-hydrogen atoms. Hydrogen atoms are represented as small spheres with arbitrary radius.

**Fig. 3.**
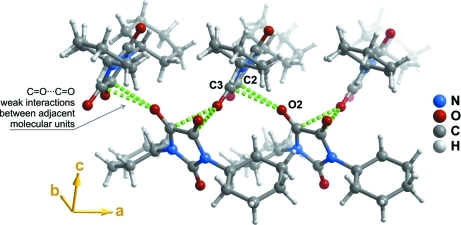
Schematic representation of the weak C═O···C═O interactions (dashed green lines) connecting adjacent molecular units.

**Fig. 4.**
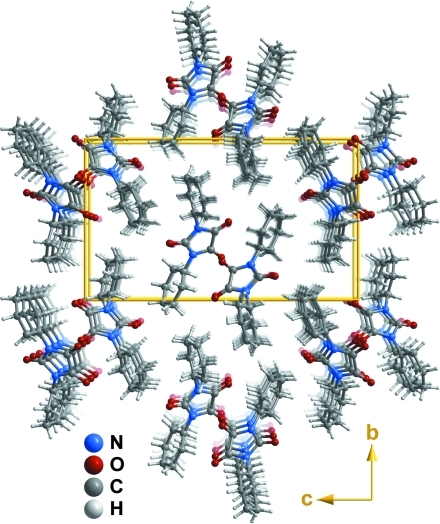
Perspective view of the crystal packing of the title compound viewed along the [100] direction of the unit cell.

## supplementary crystallographic information

### Comment

From the old literature we emphasize a handful of descriptions reporting the synthesis of parabanic acid (imidazolidine-2,4,5-trione, 1, Fig. 1) and derivatives. Among the reported synthetic methodologies, this heterocyclic compound can be prepared by the condensation of urea with diethyl oxalate in an ethanolic solution of sodium ethoxide (Murray, 1957; 1963). The synthesis of 1,3-disubstituted parabanic acid derivatives have been reported in a similar fashion, starting from 1,3-dialkylureas and following different pathways. The reaction of oxalyl chloride with 1,3-dialkylureas affords the 1,3-disubstituted parabanic skeleton upon cyclization. In other cases, the action of oxalyl chloride on carbodiimides has led to 2,2-dichloro-1,3-disubstituted imidazolidine-4,5-diones, which produced the parabanic structure after hydrolysis (Ulrichan & Sayigh, 1965). Furthermore, 3-substituted-5,5-dichlorooxazolidine-2,4-diones were obtained from the reaction of alkyl, aryl, and benzyl isocyanates with oxalyl chloride, giving in high yields the corresponding imidazolidine-2,4,5-triones after treatment with aniline (Richter *et al.*, 1984). The selectivity of the direct mono- and di-*N*-substitution of parabanic acid has also been discussed in the literature (Orazi *et al.*, 1977; Zarzyka-Niemiec & Lubczak, 2004). Concerning biological applications, several novel patented forms of parabanic acid derivatives and salts have shown interesting activities such as human AMPK activating, blood glucose-lowering and *in vivo* lipid-lowering activities. In this context, several therapeutic agents containing these compounds as the active principle are, for example, useful drugs in the treatment of diabetic complications (Sato *et al.*, 2011; Kotani *et al.*, 1997; Ishii *et al.*, 1991). In the present study, we describe the crystal structure of 1,3-dicyclohexylparabanic acid (3) (Fig. 1) (Ulrichan & Sayigh, 1965) which has been isolated *via* a completely different procedure which consists of an oxidative cleavage of the C2'—C5 single bond of 1,3-dicyclohexyl-(3-oxo-2,3-dihydrobenzofuran-2-yl)imidazolidine-2,4-dione (2) (Fig. 1), previously prepared in a two-step reaction involving the action of dicyclohexylcarbodiimide (DCC) on chromone-2-carboxylic acid (Talhi *et al.*, unpublished data).

The title compound (3) has recently been prepared and tested against cell lines modeling amyotrophic lateral sclerosis (Xia *et al.*, 2011), but its crystal structure remains unpublished. Following our interest on the structural features of compounds with biological activity (Fernandes *et al.*, 2010, 2011; Loughzail *et al.* 2011) here we wish to report the crystal structure of (3).

The asymmetric unit comprises a whole molecule (3, Fig. 2). The two cyclohexane substituent groups appear to exhibit chair conformations and their medium planes are almost perpendicular (*ca* 81 and 87°) with the medium plane of the central imidazolidine ring. The crystal packing is mainly driven by the need to effectively fill the available space in conjunction with several weak interactions, namely C═O···C═O: one O2 atom interacts with two vicinal carbonyl carbon atoms (C2 and C3) of a neighboring molecule [d_O···C_ of 2.814 (10) and 2.871 (11) Å, dashed green lines in Fig. 3]. These weak interactions contribute to the formation of a zigzag columnar arrangement of the molecular units parallel to the *a* axis of the unit cell. Columns close pack in the *bc* plane in a typical brick-wall type fashion (Fig. 4).

### Experimental

NMR spectra were recorded on a Bruker Avance 300 spectrometer (300.13 for ^1^H and 75.47 MHz for ^13^C), with CDCl_3_ used as solvent. Chemical shifts (δ) are reported in p.p.m. and coupling constants (*J*) in Hz. The internal standard was TMS. Unequivocal ^13^C assignments have been performed with the aid of two-dimensional HSQC and HMBC experiments (delays for one bond and long-range *J*_C/H_ couplings were optimized for 145 and 7 Hz, respectively).

All chemicals were purchased from commercial sources and used as received. 1,3-Dicyclohexyl-(3-oxo-2,3-dihydrobenzofuran-2-yl)imidazolidine-2,4-dione (2) was prepared according to the literature (Talhi *et al.*, 2011).

Iodine (8.63 mg, 0.034 mmol dissolved in 1 ml of DMSO) was added to a solution of 2 (0.27 g, 0.681 mmol) in DMSO (2 ml). The reaction was refluxed in a sand bath for 30 minutes. After this period, the solution was poured into ice (5 g) and water (10 ml), leading to the formation of a yellow precipitate. The solid was collected by filtration, washed with water and dissolved in dichloromethane (30 ml). This organic solution was washed with a saturated sodium thiosulfate solution (2 × 200 ml) and finally purified by silica gel column chromatography using dichloromethane as eluent. The resulting compound was recrystallized from ethanol to give bright-yellow crystals of the title compound  (Richter *et al.*, 1984).

1,3-Dicyclohexylimidazolidine-2,4,5-trione, 3, C_15_H_22_N_2_O_3_ MW: 278.35 (0.033 g, yield 17 °). ^1^H NMR (300.13 MHz, CDCl_3_): δ = 1.24-2.28 (m, 20 H, —CH_2_—, H-2', H-2'', H-3', H-3'', H-4', H-4''), 4.00 (tt, *J* = 12.0, 3.7 Hz, 2H, H-1', H-1'') ppm. ^13^C NMR (75.47 MHz, CDCl_3_): δ = 25.1 (C-4', C-4''), 25.6 (C-3', C-3''), 29.5 (C-2', C-2''), 52.2 (C-1', C-1''), 153.3 (C-4, C-5), 153.8 (C-2) ppm.

### Refinement

Hydrogen atoms bound to carbon were placed in idealized positions with C—H = 1.00 (for methine-H) and 0.99 Å (for methylene-H). These atoms were included in the final structural model in riding-motion approximation with the isotropic thermal displacement parameters fixed at 1.2×*U*_eq_ of the carbon atom to which they are attached.

The cyclohexane rings are severely affected by disorder. Attempts to model this disorder proved to be unsuccessful hence, the large electron residual density surrounding these moieties: the largest peak and hole, 0.74 and -0.42 e^.^Å^-3^, are located at 0.92 and 0.40 Å, respectively, from the C10 atom.

In the absence of significant anomalous scattering effects, 1098 Friedel pairs were averaged in the final refinement.

### Crystal data

**Table:**

C_15_H_22_N_2_O_3_	*F*(000) = 600
*M**_r_* = 278.35	*D*_x_ = 1.256 Mg m^−^^3^
Orthorhombic, *P*2_1_2_1_2_1_	Mo *K*α radiation, λ = 0.71073 Å
Hall symbol: P 2ac 2ab	Cell parameters from 1312 reflections
*a* = 6.5539 (8) Å	θ = 2.7–19.0°
*b* = 11.5029 (15) Å	µ = 0.09 mm^−^^1^
*c* = 19.524 (3) Å	*T* = 150 K
*V* = 1471.9 (3) Å^3^	Block, yellow
*Z* = 4	0.05 × 0.03 × 0.02 mm

### Data collection

**Table:**

Bruker X8 KappaCCD APEXII diffractometer	1558 independent reflections
Radiation source: fine-focus sealed tube	1028 reflections with *I* > 2σ(*I*)
graphite	*R*_int_ = 0.047
ω and φ scans	θ_max_ = 25.4°, θ_min_ = 3.6°
Absorption correction: multi-scan (*SADABS*; Sheldrick, 1997)	*h* = −7→7
*T*_min_ = 0.996, *T*_max_ = 0.998	*k* = −13→10
8292 measured reflections	*l* = −23→23

### Refinement

**Table:**

Refinement on *F*^2^	Secondary atom site location: difference Fourier map
Least-squares matrix: full	Hydrogen site location: inferred from neighbouring sites
*R*[*F*^2^ > 2σ(*F*^2^)] = 0.106	H-atom parameters constrained
*wR*(*F*^2^) = 0.320	*w* = 1/[σ^2^(*F*_o_^2^) + (0.1747*P*)^2^ + 2.7021*P*] where *P* = (*F*_o_^2^ + 2*F*_c_^2^)/3
*S* = 1.06	(Δ/σ)_max_ < 0.001
1558 reflections	Δρ_max_ = 0.74 e Å^−^^3^
181 parameters	Δρ_min_ = −0.42 e Å^−^^3^
72 restraints	Absolute structure: nd
Primary atom site location: structure-invariant direct methods	

### Special details

**Table:**

Geometry. All e.s.d.'s (except the e.s.d. in the dihedral angle between two l.s. planes) are estimated using the full covariance matrix. The cell e.s.d.'s are taken into account individually in the estimation of e.s.d.'s in distances, angles and torsion angles; correlations between e.s.d.'s in cell parameters are only used when they are defined by crystal symmetry. An approximate (isotropic) treatment of cell e.s.d.'s is used for estimating e.s.d.'s involving l.s. planes.
Refinement. Refinement of *F*^2^ against ALL reflections. The weighted *R*-factor *wR* and goodness of fit *S* are based on *F*^2^, conventional *R*-factors *R* are based on *F*, with *F* set to zero for negative *F*^2^. The threshold expression of *F*^2^ > σ(*F*^2^) is used only for calculating *R*-factors(gt) *etc*. and is not relevant to the choice of reflections for refinement. *R*-factors based on *F*^2^ are statistically about twice as large as those based on *F*, and *R*- factors based on ALL data will be even larger.

### Fractional atomic coordinates and isotropic or equivalent isotropic displacement parameters (Å^2^)

**Table:**

	*x*	*y*	*z*	*U*_iso_*/*U*_eq_	
N1	0.2051 (11)	0.2826 (7)	0.6061 (4)	0.0494 (19)	
N2	0.3789 (12)	0.4457 (7)	0.5865 (5)	0.061 (2)	
O1	0.4234 (13)	0.3500 (10)	0.6889 (4)	0.103 (4)	
O2	0.0437 (9)	0.2643 (6)	0.5014 (4)	0.0559 (17)	
O3	0.2667 (13)	0.4828 (6)	0.4759 (4)	0.070 (2)	
C1	0.3461 (15)	0.3562 (10)	0.6341 (5)	0.056 (3)	
C2	0.1559 (11)	0.3133 (7)	0.5403 (4)	0.0366 (18)	
C3	0.2743 (14)	0.4269 (7)	0.5262 (5)	0.046 (2)	
C4	0.128 (2)	0.1759 (12)	0.6392 (7)	0.092 (4)	
H4	0.0608	0.1463	0.5967	0.110*	
C5	−0.0635 (16)	0.1903 (9)	0.6766 (5)	0.061 (3)	
H5A	−0.1679	0.2213	0.6448	0.073*	
H5B	−0.0430	0.2486	0.7133	0.073*	
C6	−0.143 (2)	0.0794 (15)	0.7083 (7)	0.101 (4)	
H6A	−0.1945	0.0989	0.7545	0.122*	
H6B	−0.2615	0.0539	0.6808	0.122*	
C7	−0.014 (3)	−0.0147 (13)	0.7150 (8)	0.107 (5)	
H7A	−0.0962	−0.0843	0.7036	0.128*	
H7B	0.0191	−0.0206	0.7644	0.128*	
C8	0.1714 (19)	−0.0265 (11)	0.6797 (6)	0.075 (3)	
H8A	0.1537	−0.0871	0.6442	0.091*	
H8B	0.2753	−0.0552	0.7124	0.091*	
C9	0.255 (2)	0.0814 (9)	0.6454 (6)	0.078 (3)	
H9A	0.3775	0.1063	0.6712	0.094*	
H9B	0.3010	0.0593	0.5989	0.094*	
C10	0.5293 (19)	0.5375 (11)	0.5983 (8)	0.094 (4)	
H10	0.5748	0.5134	0.6451	0.112*	
C11	0.4542 (15)	0.6494 (8)	0.6151 (5)	0.054 (2)	
H11A	0.3762	0.6429	0.6583	0.065*	
H11B	0.3568	0.6729	0.5789	0.065*	
C12	0.6092 (17)	0.7462 (9)	0.6235 (6)	0.066 (3)	
H12A	0.5468	0.8193	0.6070	0.080*	
H12B	0.6370	0.7559	0.6730	0.080*	
C13	0.8030 (18)	0.7306 (10)	0.5885 (7)	0.080 (3)	
H13A	0.7986	0.7795	0.5468	0.096*	
H13B	0.9097	0.7639	0.6185	0.096*	
C14	0.8732 (18)	0.6172 (11)	0.5677 (7)	0.083 (3)	
H14A	0.9797	0.5925	0.6006	0.099*	
H14B	0.9405	0.6258	0.5226	0.099*	
C15	0.7265 (14)	0.5231 (8)	0.5621 (5)	0.050 (2)	
H15A	0.7920	0.4513	0.5793	0.060*	
H15B	0.6970	0.5109	0.5128	0.060*	

### Atomic displacement parameters (Å^2^)

**Table:**

	*U*^11^	*U*^22^	*U*^33^	*U*^12^	*U*^13^	*U*^23^
N1	0.034 (3)	0.061 (5)	0.054 (4)	0.001 (4)	0.008 (3)	0.011 (4)
N2	0.034 (4)	0.048 (5)	0.101 (6)	−0.008 (3)	−0.002 (4)	−0.043 (5)
O1	0.077 (5)	0.183 (10)	0.049 (4)	0.053 (6)	−0.018 (4)	−0.041 (5)
O2	0.043 (3)	0.048 (4)	0.077 (4)	0.003 (3)	−0.011 (3)	−0.021 (3)
O3	0.090 (5)	0.044 (4)	0.075 (4)	0.031 (4)	0.034 (4)	0.019 (3)
C1	0.041 (5)	0.072 (7)	0.056 (6)	0.014 (5)	−0.005 (5)	−0.020 (5)
C2	0.028 (4)	0.038 (4)	0.044 (4)	0.001 (3)	−0.007 (3)	−0.002 (3)
C3	0.046 (5)	0.027 (4)	0.063 (5)	0.008 (4)	0.007 (5)	−0.006 (4)
C4	0.061 (6)	0.097 (8)	0.118 (8)	0.019 (6)	0.030 (6)	0.053 (7)
C5	0.051 (5)	0.064 (6)	0.066 (5)	−0.007 (5)	0.015 (4)	−0.017 (5)
C6	0.067 (6)	0.137 (9)	0.100 (7)	−0.004 (7)	0.025 (6)	0.043 (7)
C7	0.120 (9)	0.078 (7)	0.122 (8)	−0.027 (7)	0.034 (8)	−0.003 (7)
C8	0.073 (6)	0.081 (7)	0.073 (6)	−0.014 (6)	−0.005 (5)	0.016 (5)
C9	0.079 (7)	0.052 (6)	0.103 (7)	−0.005 (6)	0.035 (6)	−0.007 (5)
C10	0.061 (6)	0.075 (7)	0.145 (9)	−0.023 (6)	0.017 (7)	−0.046 (7)
C11	0.049 (5)	0.043 (5)	0.071 (5)	−0.005 (4)	0.013 (4)	−0.003 (4)
C12	0.062 (6)	0.056 (6)	0.080 (6)	−0.016 (5)	0.011 (5)	−0.022 (5)
C13	0.070 (6)	0.054 (6)	0.117 (7)	−0.020 (5)	0.027 (6)	−0.001 (6)
C14	0.050 (5)	0.079 (7)	0.119 (7)	−0.012 (5)	0.016 (6)	−0.021 (6)
C15	0.037 (4)	0.051 (5)	0.061 (5)	0.005 (4)	0.005 (4)	−0.011 (4)

### Geometric parameters (Å, °)

**Table:**

N1—C1	1.367 (12)	C8—C9	1.512 (16)
N1—C2	1.372 (10)	C8—H8A	0.9900
N1—C4	1.475 (14)	C8—H8B	0.9900
N2—C3	1.379 (12)	C9—H9A	0.9900
N2—C1	1.404 (14)	C9—H9B	0.9900
N2—C10	1.463 (13)	C10—C11	1.416 (15)
O1—C1	1.185 (11)	C10—C15	1.482 (15)
O2—C2	1.199 (10)	C10—H10	1.0000
O3—C3	1.176 (10)	C11—C12	1.516 (13)
C2—C3	1.544 (12)	C11—H11A	0.9900
C4—C9	1.373 (16)	C11—H11B	0.9900
C4—C5	1.464 (15)	C12—C13	1.453 (16)
C4—H4	1.0000	C12—H12A	0.9900
C5—C6	1.510 (18)	C12—H12B	0.9900
C5—H5A	0.9900	C13—C14	1.442 (16)
C5—H5B	0.9900	C13—H13A	0.9900
C6—C7	1.38 (2)	C13—H13B	0.9900
C6—H6A	0.9900	C14—C15	1.452 (14)
C6—H6B	0.9900	C14—H14A	0.9900
C7—C8	1.405 (19)	C14—H14B	0.9900
C7—H7A	0.9900	C15—H15A	0.9900
C7—H7B	0.9900	C15—H15B	0.9900
			
C1—N1—C2	111.9 (8)	H8A—C8—H8B	107.3
C1—N1—C4	124.8 (9)	C4—C9—C8	118.0 (10)
C2—N1—C4	123.0 (9)	C4—C9—H9A	107.8
C3—N2—C1	112.0 (7)	C8—C9—H9A	107.8
C3—N2—C10	125.6 (11)	C4—C9—H9B	107.8
C1—N2—C10	121.9 (10)	C8—C9—H9B	107.8
O1—C1—N1	127.8 (12)	H9A—C9—H9B	107.1
O1—C1—N2	125.2 (11)	C11—C10—N2	117.3 (10)
N1—C1—N2	107.0 (7)	C11—C10—C15	121.0 (10)
O2—C2—N1	128.1 (8)	N2—C10—C15	115.5 (9)
O2—C2—C3	126.5 (8)	C11—C10—H10	98.3
N1—C2—C3	105.5 (7)	N2—C10—H10	98.3
O3—C3—N2	130.5 (9)	C15—C10—H10	98.3
O3—C3—C2	126.1 (9)	C10—C11—C12	117.3 (9)
N2—C3—C2	103.3 (7)	C10—C11—H11A	108.0
C9—C4—C5	124.4 (10)	C12—C11—H11A	108.0
C9—C4—N1	119.4 (9)	C10—C11—H11B	108.0
C5—C4—N1	114.6 (10)	C12—C11—H11B	108.0
C9—C4—H4	94.1	H11A—C11—H11B	107.2
C5—C4—H4	94.1	C13—C12—C11	116.4 (9)
N1—C4—H4	94.1	C13—C12—H12A	108.2
C4—C5—C6	113.8 (10)	C11—C12—H12A	108.2
C4—C5—H5A	108.8	C13—C12—H12B	108.2
C6—C5—H5A	108.8	C11—C12—H12B	108.2
C4—C5—H5B	108.8	H12A—C12—H12B	107.3
C6—C5—H5B	108.8	C14—C13—C12	121.5 (9)
H5A—C5—H5B	107.7	C14—C13—H13A	106.9
C7—C6—C5	119.5 (11)	C12—C13—H13A	106.9
C7—C6—H6A	107.4	C14—C13—H13B	106.9
C5—C6—H6A	107.4	C12—C13—H13B	106.9
C7—C6—H6B	107.4	H13A—C13—H13B	106.7
C5—C6—H6B	107.4	C13—C14—C15	119.0 (9)
H6A—C6—H6B	107.0	C13—C14—H14A	107.6
C6—C7—C8	123.9 (13)	C15—C14—H14A	107.6
C6—C7—H7A	106.4	C13—C14—H14B	107.6
C8—C7—H7A	106.4	C15—C14—H14B	107.6
C6—C7—H7B	106.4	H14A—C14—H14B	107.0
C8—C7—H7B	106.4	C14—C15—C10	117.3 (8)
H7A—C7—H7B	106.4	C14—C15—H15A	108.0
C7—C8—C9	116.9 (12)	C10—C15—H15A	108.0
C7—C8—H8A	108.1	C14—C15—H15B	108.0
C9—C8—H8A	108.1	C10—C15—H15B	108.0
C7—C8—H8B	108.1	H15A—C15—H15B	107.2
C9—C8—H8B	108.1		
			
C2—N1—C1—O1	−177.2 (9)	C1—N1—C4—C5	95.6 (13)
C4—N1—C1—O1	−2.9 (15)	C2—N1—C4—C5	−90.7 (13)
C2—N1—C1—N2	5.0 (10)	C9—C4—C5—C6	−16 (2)
C4—N1—C1—N2	179.4 (8)	N1—C4—C5—C6	178.3 (11)
C3—N2—C1—O1	177.6 (9)	C4—C5—C6—C7	16 (2)
C10—N2—C1—O1	5.2 (15)	C5—C6—C7—C8	−17 (3)
C3—N2—C1—N1	−4.6 (10)	C6—C7—C8—C9	14 (2)
C10—N2—C1—N1	−177.0 (8)	C5—C4—C9—C8	15 (2)
C1—N1—C2—O2	177.1 (8)	N1—C4—C9—C8	179.9 (11)
C4—N1—C2—O2	2.6 (13)	C7—C8—C9—C4	−12.7 (19)
C1—N1—C2—C3	−3.5 (9)	C3—N2—C10—C11	82.0 (15)
C4—N1—C2—C3	−178.0 (8)	C1—N2—C10—C11	−106.7 (14)
C1—N2—C3—O3	−179.9 (9)	C3—N2—C10—C15	−70.7 (15)
C10—N2—C3—O3	−7.8 (15)	C1—N2—C10—C15	100.6 (13)
C1—N2—C3—C2	2.4 (9)	N2—C10—C11—C12	−176.7 (11)
C10—N2—C3—C2	174.4 (9)	C15—C10—C11—C12	−25.6 (18)
O2—C2—C3—O3	2.2 (13)	C10—C11—C12—C13	23.8 (16)
N1—C2—C3—O3	−177.3 (9)	C11—C12—C13—C14	−20.9 (19)
O2—C2—C3—N2	−179.9 (8)	C12—C13—C14—C15	19 (2)
N1—C2—C3—N2	0.6 (8)	C13—C14—C15—C10	−19.0 (19)
C1—N1—C4—C9	−70.9 (17)	C11—C10—C15—C14	23.2 (19)
C2—N1—C4—C9	102.8 (14)	N2—C10—C15—C14	174.9 (11)
